# The gene-reduction effect of chromosomal losses detected in gastric cancers

**DOI:** 10.1186/1471-230X-10-138

**Published:** 2010-11-20

**Authors:** Seung-Jin Hong, Eun-Jung Jeon, Jung-Hwan Oh, Eun-Joo Seo, Sang-Wook Choi, Mun-Gan Rhyu

**Affiliations:** 1 Department of Microbiology, College of Medicine, The Catholic University of Korea, Seoul, Korea; 2 Department of Internal Medicine, College of Medicine, The Catholic University of Korea, Seoul, Korea; 3 Department of Clinical Pathology, College of Medicine, The Catholic University of Korea, Seoul, Korea

## Abstract

**Background:**

The level of loss of heterozygosity (LOH) that reduces a gene dose and exerts a cell-adverse effect is known to be a parameter for the genetic staging of gastric cancers. This study investigated if the cell-adverse effect induced with the gene reduction was a rate-limiting factor for the LOH events in two distinct histologic types of gastric cancers, the diffuse- and intestinal-types.

**Methods:**

The pathologic specimens obtained from 145 gastric cancer patients were examined for the level of LOH using 40 microsatellite markers on eight cancer-associated chromosomes (3p, 4p, 5q, 8p, 9p, 13q, 17p and 18q).

**Results:**

Most of the cancer-associated chromosomes were found to belong to the gene-poor chromosomes and to contain a few stomach-specific genes that were highly expressed. A baseline-level LOH involving one or no chromosome was frequent in diffuse-type gastric cancers. The chromosome 17 containing a relatively high density of genes was commonly lost in intestinal-type cancers but not in diffuse-type cancers. A high-level LOH involving four or more chromosomes tended to be frequent in the gastric cancers with intestinal and mixed differentiation. Disease relapse was common for gastric cancers with high-level LOH through both the hematogenous (38%) and non-hematogenous (36%) routes, and for the baseline-level LOH cases through the non-hematogenous route (67%).

**Conclusions:**

The cell-adverse effect of gene reduction is more tolerated in intestinal-type gastric cancers than in diffuse-type cancers, and the loss of high-dose genes is associated with hematogenous metastasis.

## Background

Gastric cancers undergo hypermutations in simple repeat sequences and imbalanced chromosomal losses, and these genetic alterations are detected as a microsatellite instability (MSI) and loss of heterozygosity (LOH), respectively, by using highly polymorphic microsatellite markers [1-4]. The pathobiological behavior and prognosis of gastric cancers have been associated with the level of LOH and the presence of MSI [3-6]. The depth of invasion and lymph node metastasis are considerably advanced in the cases of small-sized cancers with high-level LOH, and these histologic parameters tends to be linearly related with the cancer size for the low-level LOH cases [7]. Because a single microsatellite genotype is similarly detected in the entire surgical tissue as well as the endoscopic biopsy sample [3,7], it is possible to predict the probability of disease relapse and to decide the appropriate resection procedure based on the microsatellite analysis of a pretreatment biopsy tissue.

The two-hits hypothesis has proposed that chromosomal loss and point mutation result in the biallelic inactivation of tumor suppressor genes [8]. Chromosomal losses that exert a deleterious effect on cell growth are much less tolerated than chromosomal gains, and the two genetic hits providing a growth advantage could protect cancer progenitor cells from lethal LOH events [9,10]. Additionally, previous studies have proposed that the methylated gene-control regions are decreasingly methylated in a LOH-level-dependent manner in gastric cancers [11,12] via a dosage compensation mechanism that sustains a gene or transcription dose during subsequent cell cycles [9]. With respect to the dosage compensation mechanism, chromosomal gain and LOH-associated demethylation may result from dose-compensatory genetic and epigenetic responses to a reduction in the chromosomal dose [11-14]. Therefore, the LOH events combined with a cell-adverse effect and a dose-compensatory response in addition to tumor suppressor gene inactivation are likely to determine the pathobiological behavior of cancer progenitor cells.

Gastric cancers have been categorized into two distinct histologic types, that is, the diffuse- and intestinal-types [15,16]. Diffuse-type cancers are common in young-aged patients who lack a precancerous lesion, whereas intestinal-type cancers are common in old-aged patients associated with the precancerous intestinal metaplasia that resemble intestinal glands [17]. Assuming that the marrow-derived stem cells are adapting to the stomach tissue-environment [18], the newly-fixed stem cells, which are potentially non-cohesive and invasive in young individuals, develop into diffuse-type cancer. Intestinal-type cancers are frequent among the old-aged patients who have the long-term adaptation of newly-fixed stem cells to the gastric tissue environment. In this regard, a given LOH event may affect distinct doses of transcription between diffuse- and intestinal-type gastric cancers that establish distinct gene expression patterns.

In this study, correlations were made between the LOH events and pathobiological behavior of gastric cancers in terms of the cell-adverse effects of gene reduction. The eight cancer-associated chromosomes we examined had a low density of genes and no stomach-specific genes, which could result in a mild cell-adverse effect of LOH. The LOH events were frequent in intestinal-type gastric cancers, in which the loss of a chromosome would affect a low dose of transcription and exert a mild cell-adverse effect.

## Methods

### Selection of cases

One hundred and forty-five patients with gastric cancer who underwent a curative surgical resection between February 1996 and June 2003 were enrolled in this study. The clinicopathologic and radiologic information was obtained by reviewing the detailed records of the patients. One hundred and sixteen cases were previously analyzed by multifocal genetic examination of the heterogeneous tumor sites [3]. The remaining 29 patients were examined using a single tissue block of each tumor.

The microscopic slides were reviewed and then a tumor site that was representative of the histologic feature was chosen. The histologic type of gastric cancer was defined as intestinal (glandular, cohesive or solid), diffuse, and mixed according to the Lauren classification [15,16] and the degree of differentiation was graded according to the WHO classification. The tumor locations considered were the cardia, body, and antrum. The clinicopathologic tumor stage was determined according to the Tumor-Node-Metastasis (TNM) criteria [19]. Most gastric cancer patients (140 of 145, 97%) had undergone R0 gastrectomy and D2 or more extended lymphadenectomy. An overall mean of 28.6 (median: 33) lymph nodes were removed along with the specimen. None of the patients received pre-operative chemotherapy and radiation therapy.

### Follow-up data on recurrence and survival

A combination therapy of intravenous mitomycin, fluorouracil, and cytarabine (MFC) followed by oral fluorouracil was administered as a standard postoperative adjuvant treatment according to the physician's judgment on the overall prognosis of each case. During the follow-up period, a physical examination, laboratory tests, chest radiography, abdominal ultrasonography or computed tomography, and gastrofiberscopy were carried out every 3 or 6 months and disease relapse was histologically confirmed, if possible. When more than one recurrence site was detected at the first time of failure, the individual recurrences were counted separately. The recurrence site was categorized into hematogenous metastasis involving the lung, liver, or other distant organs, and non-hematogenous metastasis that included nodal involvement and peritoneal dissemination. Gastric remnant cancers were excluded from the recurrence pattern analysis.

The overall survival time was calculated from the date of surgical resection until either the day of the last follow-up contact or cancer-related death. The data on patients who died from other causes was censored at the time of death. Statistical analysis was performed in April 2007. The mean follow-up period of all the surviving patients was 40 months (range: 5 to 96 months) and was completed by 98% of the enrolled patients. During the survival analysis, 50 patients had died as a result of their cancers and 12 patients had died of other causes.

### Tissue microdissection and DNA amplification

Five serial 7 μm-thick sections from each formalin-fixed paraffin-embedded tissue sample were deparaffinized and briefly stained with hematoxylin and eosin. A single tumor-cell-rich focus was chosen by microscopic examination and a tumor area ranging from 5 mm to 7 mm in diameter was manually dissected under a stereomicroscope (magnification, × 40) using a surgical scalpel. The microdissected tissue pieces were microscopically examined if the tumor cell content was > 70%, which had been confirmed to reflect a difference in the genetic content between the normal and tumor tissues [3,7].

Approximately 1,000 microdissected tumor cells were incubated in 20 μL of DNA extraction buffer (0.5% Tween-20, 1 mM EDTA pH 8.0, 50 mM Tris pH 8.0, 0.5 mg/mL proteinase K) at 37°C for 24 hr. Formalin-fixed paraffin-embedded tissue DNA tends to be poorly amplified by PCR in older specimens. Most DNAs were extracted within five years after the preparation of the pathological specimens, which ensures the DNA's quality for PCR. The amount of DNA, with varying qualities, of the tissue lysate was determined based on the PCR band intensity with 5-10 ng/μL of the DNA, which was amplified using a microsatellite primer set, *D19S226 *(forward: 5' - CCA GCA GAT TTT GGT GTT GTC TA - 3'; reverse: 5' - ACA GAG CCA GAG CCA GTA GGA GT - 3'; amplicon size: 164 bp). The PCR amplification was performed under a hot-start condition with using a radioisotope (α-^32^P dCTP, PerkinElmer, Boston, MA, USA) as described previously [3,7]. Briefly, a total of 10 μL PCR mixture underwent 32 cycles of a serial amplification step that consisted of 94°C for 50 sec, a primer-specific annealing temperature for 50 sec, and 72°C for 1 min. The radioisotope-labeled microsatellite sequences were separated on a 6% polyacrylamide gel that contained 7 M urea and they were visualized by repeated exposures of each autoradiograph and using a radioluminograph scanner (BAS 2500, Fuji Photo Film Co. Ltd., Kanakawa, Japan).

### Analysis of microsatellite alleles

**Figure 1:**
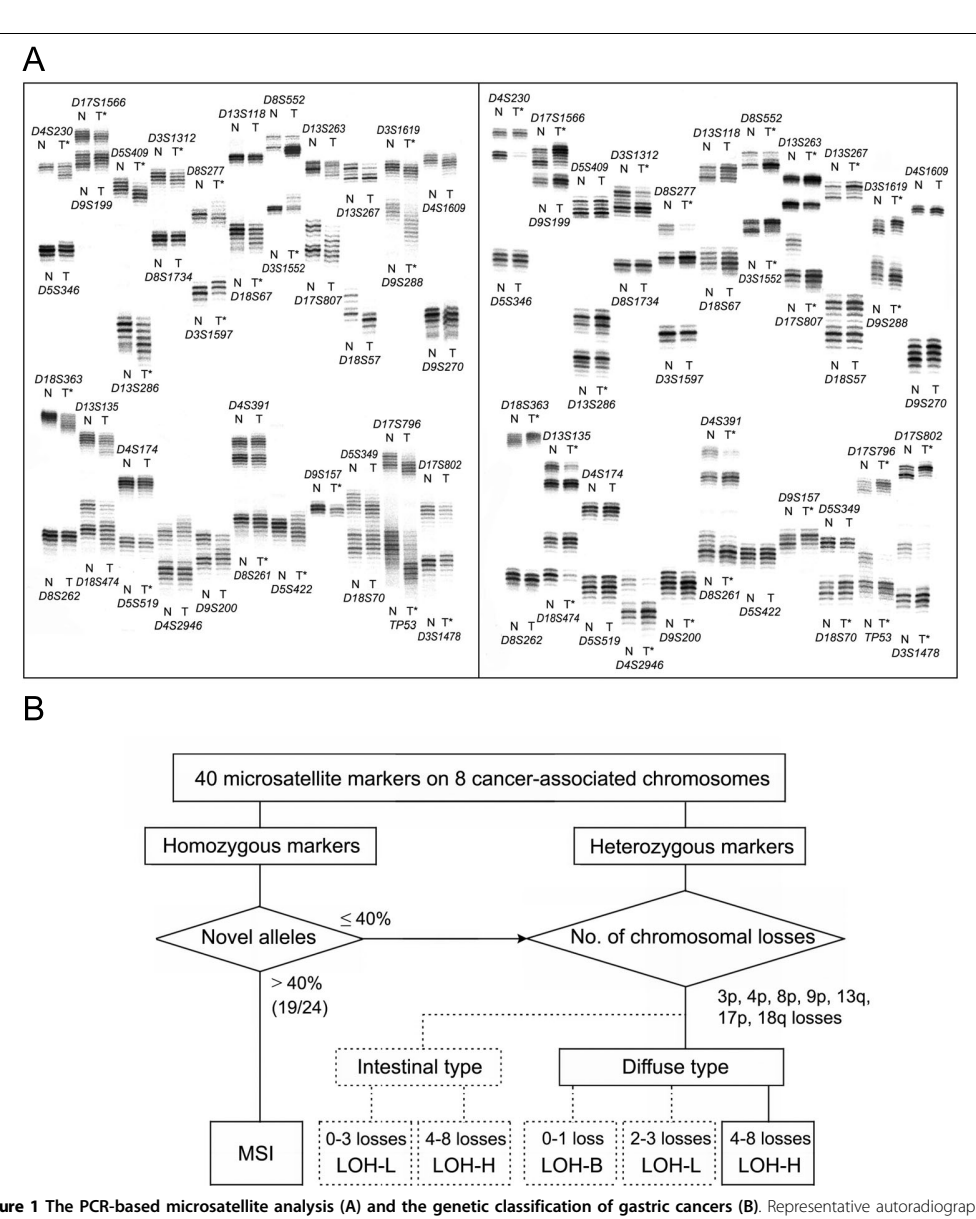
**The PCR-based microsatellite analysis (A) and the genetic classification of gastric cancers (B)**. Representative autoradiographs of the samples that were examined by PCR-based microsatellite analysis (A) and the genetic classification of the intestinal-type and diffuse-type gastric cancers based on loss of heterozygosity (LOH) and microsatellite instability (MSI) (B). (A) The left gel electrophoresis shows high-frequency MSI at more than 40% of the 15 homozygous markers. The right gel electrophoresis shows high-level LOH involving chromosomes 3p, 4p, 5q, 9p, 13q, 17p and 18q. The normal (N) and corresponding tumor (T) DNAs are indicated above each allelic band. The asterisk indicates MSI and LOH. The genomic DNA microdissected from formalin-embedded paraffin-fixed tissue was amplified and labeled by [α-32P]dCTP in the hot start condition of multiplex PCR. A total of 80 microsatellite amplicons from each specimen were run simultaneously on two sequencing gels. (B) Using a panel of 40 microsatellite markers on eight cancer-associated chromosomes, LOH was interpreted at the heterozygous markers if 40% or less of the homozygous markers exhibited MSI. The extent of the chromosomal losses, as scored according to the number of LOH-positive chromosomes, was divided into high-level (LOH-H) and low-level (LOH-L) losses for both the intestinal-and diffuse-types. In cases of diffuse type gastric cancers, zero or one chromosomal loss was classified into the baseline-level (LOH-B).

The guidelines for scoring the status of LOH and MSI have been detailed in previous studies that used the same panel of microsatellite markers [7]. As a reference type of the microsatellite alleles, we retrieved the dinucleotide repeat markers that ranged from 88 bp to 247 bp in amplicon size and that had a heterozygous frequency >50%. The highly polymorphic microsatellite markers on 8 cancer-associated chromosomes (3p, 4p, 5q, 8p, 9p, 13q, 17p and 18q), which frequently suffered from LOH in gastric cancer [3,7,20], were used to increase the number of heterozygous alleles on each arm (Figure 1A). The five microsatellite markers on each chromosomal arm showed the clear PCR bands of the heterozygous alleles and spanned the entire length of the eight chromosomes [3,5]. To ensure the chromosomal reduction, the chromosomal loss was assigned when the LOH event involved more than two microsatellite markers on one chromosomal arm [13]. Forty pairs of microsatellite primers were mixed in a total of 22 reaction tubes and each of which contained one (4 mixtures) or two (18 mixtures) primer sets that spanned different-sized amplicons at the same annealing temperatures. The resultant unequivocal microsatellite bands indicated the high specificity of the multiplex PCR condition, which was useful for small amounts of microdissected pathologic tissues.

Based on the same classification of the microsatellite genotypes that was applied in the previous study (Figure 1B) [7], the allelic profiles of the 40 microsatellite markers were analyzed for MSI at the homozygous markers that showed a few shadow or stutter bands in a pair of normal and tumor DNAs. Because MSI obscures the heterozygous allelic status, the allelic profiles of the 40 microsatellite sequences were initially analyzed for MSI at the homozygous markers. The LOH status was determined based on the allelic loss in the heterozygous marker (Figure 1B). The extent of chromosomal losses in each case was scored according to the number of constitutional chromosomal losses involving more than one microsatellite allele.

### Analysis of *in-silico *data for the gene density and transcription of individual chromosomes

A total of 17,723 reference genes identified in a public database (http://genome.ucsc.edu/, March 2006 assembly) [21] were analyzed to calculate the number of genes per 1-Mb nucleotides segment. Serial analysis of gene expression (SAGE) data of normal gastric mucosa was obtained from a public database (http://www.ncbi.nlm.nih.gov/geo/, "SAGE_Stomach_normal_B_antrum"). The transcriptional activity of individual genes was calculated by combining the reference gene map and the expressed gene tags. Based on a comparison of the microarray and SAGE data evaluating the gene expression profiles, the number of transcripts counted in the SAGE data was found to accurately estimate a great difference in the gene activity between the stomach-specific genes and housekeeping genes [21].

### Statistical analysis

Fisher's exact test and *χ*^2 ^tests were used to compare the clinicopathologic features with the microsatellite genotype of the gastric cancers. Probability curves were calculated according to the Kaplan-Meier method and compared using the log-rank test. Multivariate analysis was performed by the Cox's proportional hazards method with using stepwise procedures. Probability values were two-tailed, with a *P *value less than 0.05 being regarded as statistically significant. The statistical software package SPSS 11.0 (SPSS Inc., Chicago, IL, USA) was used for data analysis.

## Results

### Analysis of the LOH events in gastric cancers

Most of the gastric cancers (116 out of 145 cases) were examined in a previous multifocal analysis on the heterogeneous tumor sites from a given gastric cancer [3]. Ninety five percent of the gastric cancers examined were found to have either a similar level of LOH or MSI commonly shared by heterogeneous tumor sites and then each gastric cancer was categorized into a single microsatellite genotype, and not a mixed genotype. The microsatellite genotypes of the 27 additional cases were scored according to the level of LOH and the presence of MSI, which were detected in a single tissue piece containing a representative histological component.

Each chromosomal loss was correlated with the clinicopathologic parameters of the gastric cancers (Table 1). Most chromosomal losses (4p, 5q, 9p, 13q, 17p and 18q) were associated with late-onset disease (*P *< 0.05). Two or more chromosomal losses were concurrently related with the antral location (9p and 18q losses), the intestinal-or mixed-type histology (5q, 17p and 18q losses), and a poor survival (3p, 9p and 13q losses) (*P *< 0.05). The 17p loss was most frequent in the intestinal-type cancers (67%). The 13q loss was most frequent in the diffuse-type cancers (47%).

**Table 1:** Relationships of the clinicopathologic characteristics and single chromosomal loss

Characteristics		3p loss	4p loss	5q loss	8p loss	9p loss	13q loss	17p loss	18q loss
No. of patients	130	47	47	51	30	50	57	77	55
Age									
Mean	60	61	63	63	63	64	63	63	63
± SD	± 12.60	± 10.18	± 10.49	± 9.28	± 10.24	± 10.28	± 10.43	± 10.94	± 11.20
*P*		0.290	**0.029**	**0.014**	0.102	**0.003**	**0.030**	**< 0.0001**	**0.030**
Sex									
Male	86	37	34	36	18	37	42	52	36
Female	44	10	13	15	12	13	15	25	19
*P*		**0.033**	0.335	0.450	0.510	0.182	0.136	0.710	1.000
Tumor location									
Cardia	13	4	3	5	1	2	5	8	3
Body	48	16	16	13	10	14	16	24	16
Antrum	69	27	28	33	19	34	36	45	36
*P*		0.743	0.424	0.078	0.267	**0.018**	0.119	0.253	**0.043**
Histologic type									
Intestinal	54	19	19	23	13	22	20	36	27
Diffuse	32	9	9	6	5	8	15	11	7
Mixed	44	19	19	22	12	20	22	30	21
*P*		0.395	0.395	**0.018**	0.480	0.176	0.404	**0.004**	**0.026**
Tumor size									
Mean	5.3	5.8	5.1	4.9	5.1	4.9	5.7	5.2	5.5
± SD	± 3.01	± 2.86	± 2.64	± 2.29	± 2.42	± 2.73	± 3.02	± 2.86	± 3.13
*P*		0.129	0.631	0.131	0.705	0.181	0.179	0.564	0.483
Stage									
I & II	64	20	21	20	14	22	21	36	24
III & IV	66	27	26	31	16	28	36	41	31
*P*		0.277	0.469	0.075	0.836	0.372	**0.014**	0.593	0.292
Recurrence									
Peritoneal	24	10	9	8	5	10	12	16	11
Lymph node	10	4	3	3	2	5	4	6	3
Hematogenous	22	13	11	14	8	11	14	15	10
Total	56	27	23	25	15	26	30	37	24
*P*		0.422	0.507	0.070	0.428	0.826	0.415	0.899	0.663
Vital status	119	42	41	47	29	44	51	68	48
Alive	70	19	21	26	16	20	24	36	27
Dead	49	23	20	21	13	24	27	32	21
*P*		**0.021**	0.152	0.330	0.402	**0.019**	**0.019**	0.094	0.389

*P *values less than 0.05 are indicated in boldface print.

**Figure 2:**
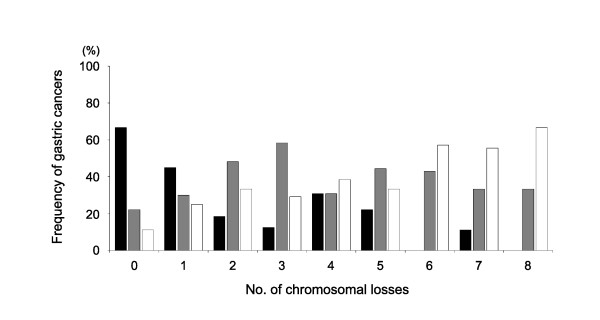
**Relationships between the histologic type and no. of chromosomal losses in the gastric cancers**. The histologic type of gastric cancer was defined as the diffuse- (n = 32, closed box), intestinal- (n = 54, gray box), and mixed-types (n = 44, open box), according to Lauren classification.

A large fraction (52%) of diffuse-type gastric cancers had one or no chromosomal losses, which were classified into a baseline-level LOH (LOH-B), because most of the intestinal-and mixed-type cancers (86%) had two or more chromosomal losses (Figure 2) [7]. The high-level LOH cases with four or more chromosomal losses were most frequent in the mixed differentiation cases with both intestinal- and diffuse-type gastric cancers (44%). Overall, 15 LOH-B (10%), 65 LOH-L (45%), 50 LOH-H (35%), and 15 MSI (10%) cases were identified in the 145 surgical specimens.

### Analysis of the gene density and the highly expressed genes on cancer-associated chromosomes

The density of genes per 1-Mb segment, the top 100 active genes that were highly expressed in the stomach, and the frequency of the LOH genotypes detected in gastric cancers were analyzed on each chromosome (Table 2). In comparison of the eight cancer-associated chromosomes we examined and the remaining 14 chromosomes, the density of genes (4.8 genes versus 6.9 genes per 1-Mb segment) and the average number of the highly expressed genes (3.3 genes versus 5.3 genes) were lower in the cancer-associated chromosomes than in the other chromosomes. The mean number of transcripts estimated in each highly expressed gene was also low in the cancer-associated chromosomes (54 versus 69 transcripts per each gene). The individual autosomes were grouped in the order of gene densities. The chromosomes 2, 3, 4, 5, 8, 13 and 18 were categorized into the low-gene-density group of less than five genes per 1 Mb segment. The chromosomes 11, 16, 17, 19, 20 and 22 had a high density of genes of more than eight genes per 1 Mb segment. Consequently, of the eight cancer-associated genes that were examined, six (chromosomes 3, 4, 5, 8, 13 and 18) belonged to the gene-poor chromosomes, and the remaining two genes belonged to the intermediate-(the chromosome 9) and high-(the chromosome 17) gene-density groups. The chromosomes 17 and 13 contained high and low densities of genes (13.4 and 2.7 genes per 1-Mb segment), respectively. Chromosome 17 showed a high frequency of LOH in the LOH-L (49%) but not in the LOH-B cases (13%). The frequency of chromosome 13 loss was similar in the LOH-L (25%) and LOH-B cases (27%).

**Table 2:** The gene density, highly expressed genes, and LOH level on individual chromosomes estimated in stomach cancerous and noncancerous tissues

	No. of genes per a 1-Mb segment*	Top 100 highly expressed genes (No. of transcripts per gene)*	**Level of LOH (%)**^**§**^		
			
			Baseline (n = 15)	Low (n = 65)	High (n = 50)
Cancer-associated chromosomes					
3	4.9	3 (76)	1 (7)	18 (28)	28 (56)
4	3.6	5 (53)	2 (13)	12 (18)	33 (66)
5	4.4	4 (44)	0 (0)	14 (22)	37 (74)
8	4.1	3 (32)	0 (0)	9 (14)	21 (42)
9	5.2	4 (60)	1 (7)	15 (23)	34 (68)
13	2.7	2 (121)	4 (27)	16 (25)	37 (74)
17	13.4	5 (42)	2 (13)	32 (49)	43 (86)
18	3.2	0 (0)	1 (7)	18 (28)	36 (72)
			
Mean values	4.8	3.3 (54)			

Other chromosomes					
1	7.7	8 (47)			
2	4.7	9 (132)			
6	5.8	7 (39)			
7	5.2	6 (45)			
10	5.0	2 (37)			
11	8.9	9 (85)			
12	7.3	10 (62)			
14	5.3	6 (43)			
15	5.3	2 (90)			
16	8.6	3 (34)			
19	19.8	8 (61)			
20	8.3	2 (76)			
21	5.1	1 (268)			
22	8.4	1 (55)			
			
Mean values	6.9	5.3 (69)			

*The number of transcripts of 17,723 genes was analyzed using the previously published data [21].

^§^The details are described in the material and methods section.

### Relationships between the clinicopathologic features and the microsatellite genotypes

The 145 gastric cancers were analyzed for the relationships between the level of LOH and the clinicopathologic features (Table 3). Both the LOH-H and LOH-B gastric cancers were commonly associated with high-risk phenotypes such as lymphatic invasion (*P *= 0.006), venous invasion (*P *= 0.005), advanced stages (*P *< 0.0001), recurrence (*P *= 0.023) and a poor survival rate (*P *< 0.0001), whereas the LOH-L and MSI gastric cancers were correlated with well and moderate differentiation (*P *= 0.033) and an early tumor stage (*P *< 0.0001). The two genotypes related with high-risk-phenotype were dissimilar according to the anatomical site of occurrence and the pattern of recurrence. The LOH-H cases frequently occurred in the antral portion (70%) and they recurred through hematogenous spreading to the liver, lung and other distant organs (38%), as well as non-hematogenous metastasis such as peritoneal seeding and nodal involvement (36%). Most of the LOH-B cases were present in the cardiac and body portion (94%) and they relapsed in the peritoneal cavity (67%), rather than in distant organs (13%). The onset age of the LOH-B (45 years) and LOH-H (65 years) cases was significantly different (*P *< 0.0001).

**Table 3:** Relationships between the microsatellite genotypes and the clinicopathologic features of gastric cancers

Characteristics		High-risk genotypes			Low-risk genotypes			*P *value
						
	(%)	LOH-H (%)*	LOH-B (%)*	*P *value	LOH-L (%)*	MSI (%)*	*P *value	
No. of patient	145	50	15		65	15		
Age (year)				**< 0.0001**			**0.038**	0.710
Mean	61	65	45		60	67		
SD	± 12.5	± 9.8	± 12.7		± 11.6	± 9.8		
Sex				0.118			**0.008**	0.393
Male	90 (62)	36 (72)	7 (47)		43 (66)	4 (27)		
Female	55 (38)	14 (28)	8 (53)		22 (34)	11 (73)		
Tumor size				0.247			0.502	0.394
Mean	5.34	5.3	6.34		5.0	5.6		
SD	± 2.94	± 2.62	± 3.77		± 3.10	± 2.33		
Differentiation				0.052			0.094	**0.033**
Well	11 (8)	3 (6)			8 (12)			
Moderate	53 (36)	17 (34)	1 (7)		25 (39)	10 (67)		
Poor	81 (56)	30 (60)	14 (93)		32 (49)	5 (33)		
Tumor location				**< 0.0001**			**0.037**	0.791
Cardia	13 (9)	3 (6)	4 (27)		6 (9)			
Body	50 (35)	12 (24)	10 (67)		26 (40)	2 (13)		
Antrum	82 (56)	35 (70)	1 (6)		33 (51)	13 (87)		
Lauren classification				**< 0.0001**			**0.016**	**< 0.0001**
Intestinal	58 (40)	19 (38)	0 (0)		35 (54)	4 (27)		
Diffuse	32 (22)	9 (18)	15 (100)		8 (12)	0 (0)		
Mixed	55 (38)	22 (44)	0 (0)		22 (34)	11 (73)		
Growth pattern				0.600			**< 0.0001**	**< 0.0001**
Expanding	20 (14)	1 (2)	1 (7)		9 (14)	9 (60)		
Infiltrative	70 (48)	32 (64)	10 (67)		28 (43)			
Mixed	55 (38)	17 (34)	4 (26)		28 (43)	6 (40)		
Lymphatic invasion				1.000			0.771	**0.006**
No	44 (30)	9 (18)	3 (20)		34 (52)	5 (33)		
Yes	101 (70)	41 (82)	12 (80)		31 (48)	10 (67)		
Venous invasion				0.351			0.113	**0.005**
No	112(77)	35 (70)	8 (53)		54(83)	15 (100)		
Yes	33 (23)	15 (30)	7 (47)		11 (17)			
Tumor stage				0.757			0.763	**< 0.0001**
Early	75 (52)	16 (32)	6 (40)		42 (65)	11 (73)		
Advanced	70 (48)	34 (68)	9 (60)		23 (35)	4 (27)		
Recurrence				**0.035**			0.517	**0.023**
Non-hematogenous	36 (62)	18 (36)	10 (67)		14 (22)	2 (13)		
Hematogenous	22 (38)	19 (38)	2 (13)		3 (5)			
Vital status	130			1.000			1.000	**< 0.0001**
Alive	80 (61)	14 (32)	4 (27)		52 (87)	10 (91)		
Dead	50 (39)	30 (68)	11 (73)		8 (13)	1 (9)		

*P *values less than 0.05 are indicated in boldface print.

*The gastric cancers were categorized into high-level LOH (LOH-H), baseline-level LOH (LOH-B), low-level LOH (LOH-L) and microsatellite instability (MSI). The details are described in the material and methods section.

Gastric cancers with the MSI genotype were positively correlated with late-onset disease (67 *vs*. 60 years, *P *= 0.038), female patients (73% *vs*. 34%, *P *= 0.008), intestinal- and mixed-type cancers (100% *vs*. 75%, *P *= 0.016) and an antral location (87% *vs*. 51%, *P *= 0.037), as compared with the LOH-L cases (Table 3). Favorable tumor behaviors such as an expanding and mixed growth pattern (100% *vs*. 57%), no venous invasion (100% *vs*. 83%) and good survival (91% *vs*. 87%) were more closely associated with the MSI genotype than with the LOH-L genotype.

### Progression and recurrence of the microsatellite genotypes in relation to tumor size

**Figure 3:**
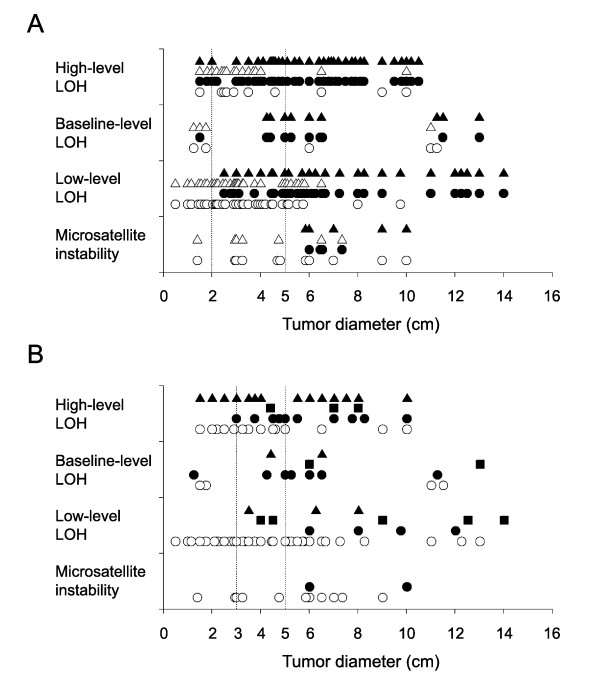
**Relationships between the tumor size and tumor progression (A) and recurrence (B) in gastric cancers that were classified into the four microsatellite genotypes**. Each genotype group is divided according to the depth of invasion (T1-2, △; T3-4, ▲), nodal involvement (N0, ○; N1-3, ●) (A), and recurrence pattern (hematogenous, ▲; lymph node, ■; peritoneal, ●; no recurrence, ○) (B). The details of genotype classification are described in the material and methods section. The vertical dotted lines indicate the cutoff tumor diameter.

The gastric cancers were analyzed for the progression (Figure 3A) and recurrence patterns (Figure 3B) of the microsatellite genotypes in relation to the tumor size. Small gastric cancers ≤ 2 cm in diameter with LOH-H (5 cases) and LOH-B (3 cases) often developed extraserosal invasion (two LOH-H), nodal metastasis (one LOH-B and two LOH-H), hematogenous recurrence (two LOH-H) and peritoneal seeding (one LOH-B). All the small gastric cancers ≤ 2 cm in diameter with LOH-L (9 cases) and MSI (1 case) were free of extraserosal invasion, lymph node metastasis and disease relapse. The LOH-L (21 cases) and MSI (3 cases) gastric cancers ≤ 3 cm in diameter showed no recurrence. Extraserosal invasion (LOH-L, 20 cases, 31%; MSI, 6 cases, 40%) and lymph node metastasis (LOH-L, 22 cases, 34%; MSI, 4 cases, 27%) were frequently detected in the large-sized LOH-L and MSI gastric cancers ≥ 5 cm in diameter.

### Prognostic implication of the three levels of chromosomal losses and MSI

**Figure 4:**
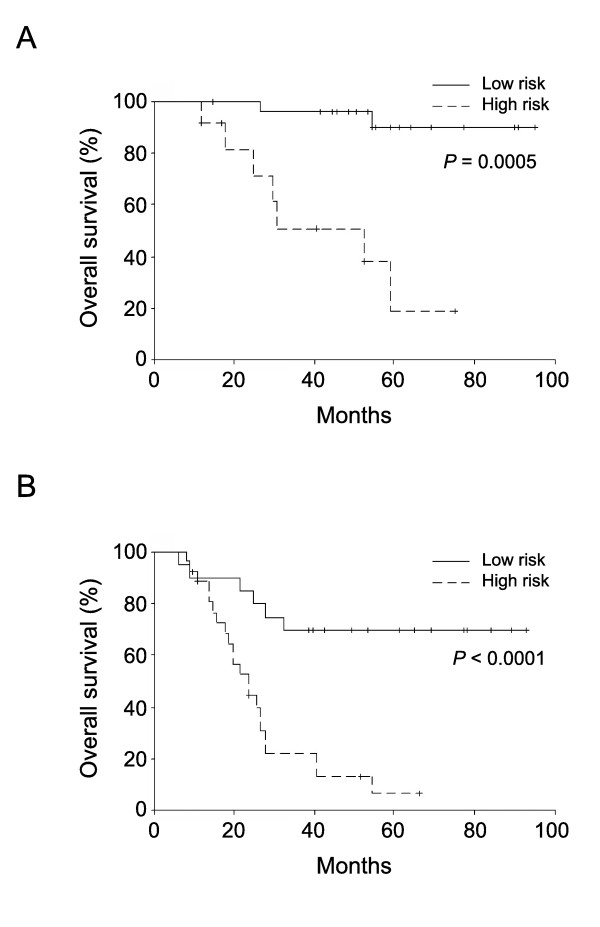
**The Kaplan-Meier survival curves of 41 stage II gastric cancer cases (A) and 52 stage III gastric cancer cases (B)**. The patients with stage II (A) or stage III (B) tumor were stratified according to the low-risk (MSI and low-level LOH) and high-risk (high-level LOH and the diffuse-type specific baseline-level LOH) genotypes. The details of genotype classification are described in the material and methods section. Log-rank tests were performed to statistically analyze the differences in patient survival.

The LOH-H and diffuse-type LOH-B genotypes were significantly associated with poor clinicopathological features when compared with LOH-L and MSI cases (Table 3). The gastric cancers were categorized into low-risk (MSI and LOH-L) and high-risk (LOH-H and diffuse-type LOH-B) tumor according to the relationships between the microsatellite genotypes and the clinicopathologic features. The Kaplan-Meier survival curves and the log-rank analysis demonstrated that the low- and high-risk genotypes were significantly associated with good and poor survivals in both stage II (*P *= 0.0005) and stage III (*P *< 0.0001) gastric cancers (Figure 4). The Cox's proportional hazards models that disclosed the microsatellite genotypes were the most significant prognostic factor (Table 4): the hazards ratios for cancer-related deaths in the patients with high-risk genotype cancer *vs*. the low-risk genotype cancer were 22.077 for stages II and III (model 1; 95% confidence interval, 6.57-74.18; *P *< 0.0001). The high-risk and low-risk genotypes were significant independent factors of survival in stage II (model 3; 15.42, 95% confidence interval, 1.71-139.5; *P *= 0.015) and stage III (model 5; 19.69, 95% confidence interval, 4.38-88.53; *P *< 0.0001). When removing the microsatellite genotypes from the Cox's models, the cancer stage, the tumor size, the growth pattern and venous invasion were the independent prognostic factors (models 2, 4 and 6).

**Table 4:** Multivariate analysis of 145 patients with gastric cancer for determining the significant prognostic factors

Category	Hazard ratio	95% CI	*P *value
Model 1 (stage II and III); Genotype variables			
High- vs. low-risk genotype	22.077	6.57 - 74.18	< 0.0001
Stage II vs. III	3.316	2.03 - 5.42	< 0.0001
Vein invasion, no vs. yes	0.440	0.23 - 0848	0.014
Age	1.001	0.98 - 1.03	0.957
Sex	0.852	0.44 - 1.67	0.640

Model 2 (stage II and III); No genotype variables			
Stage II vs. III	4.310	2.60 - 7.13	< 0.0001
Growth pattern,	0.581	0.38 - 0.90	0.014
Sex	2.079	1.02 - 4.24	0.044
Age	0.989	0.97 - 1.02	0.370

Model 3 (stage II); Genotype variables			
High- vs. low-risk genotype	15.42	1.71 - 139.5	0.015

Model 4 (stage II); No genotype variables			
Tumor size	0.050	0.01 - 0.65	0.022
Histological type	0.167	0.04 - 0.79	0.024

Model 5 (stage III); Genotype variables			
High- vs. low-risk genotype	19.69	4.38 - 88.53	< 0.0001
Tumor size	1.22	1.02 - 1.46	0.028

Model 6 (stage III); No genotype variables			
Vein invasion, no vs. yes	0.30	0.13 - 0.70	0.006

Hazard ratios were estimated in a multivariate analysis using a stepwise procedure. A variable in the model was entered when it was a significant independent factor (*P *< 0.05). Hazard ratios less than 1.00 represent a decreased risk of death, whereas hazard ratios greater than 1.00 represent an increased risk of death.

CI, confidence interval.

## Discussion

The cell-adverse effect of chromosomal loss is considered principally as a rate-limiting factor for the occurrence of LOH. For example, the postnatal survival for monosomy 22q11 (DiGeorge syndrome) [22,23] is a function of the deletion of the smallest autosome causing a minimal reduction in the chromosomal dose. Most of the cancer-associated chromosomes examined in this study were found to belong to the gene-poor chromosomes and to contain fewer stomach-specific genes that were highly expressed (Table 2). This indicates that the LOH events tend to target the chromosomes with a low dose of genes and transcription. Chromosome 17, which contained a relatively high density of genes and the *P53 *gene, was commonly lost in the intestinal- and mixed-type gastric cancers with a high-level LOH. Meanwhile, chromosome 13, which contained a low density of genes and the *RB1 *gene, was most frequently lost in the early-onset diffuse-type cancers with a baseline-level LOH (Table 2) [3]. Therefore, the adverse effect of LOH was found to be more tolerated in intestinal-type cancers than in diffuse-type cancers.

The pluripotent mesenchymal stem cells have a high number of active CpG-island genes dispersed on the entire chromosomes, whereas lineage-committed somatic cells establish a high expression of several tissue-specific genes localized on some chromosomes [21]. Given that newly fixed stem cells in young-aged individuals tend to preserve the mesenchymal cell traits, the LOH event in young-aged gastric cancer patients can exert a severe adverse effect by affecting numerous active genes. This explains the reason why the baseline-level LOH cases are frequent in diffuse-type gastric cancers in young-aged patients [3,4,7]. The LOH event in old-aged patients may exert a mild adverse effect at a late adaptation stage of newly fixed stem cells that establish a high expression of several stomach-specific genes and the down-regulation of housekeeping genes. This provides a plausible explanation for the reason why the high-level LOH cases are related with intestinal-type gastric cancers common in the old-aged patients.

A comparative analysis of stomach, colon, head, neck and thyroid cancers has described an association between the level of LOH and the expression of tissue-specific genes lacking CpG-islands [11]. The LOH events are more frequent in gastric cancers along with a higher expression of stomach-specific genes lacking CpG-islands in the gastric normal mucosa, when compared with other tissue types. This indicates a mild adverse effect of LOH events on somatic tissues that sustain a high expression of tissue-specific genes, which allow a high-level of LOH. In this regard, the occurrence of a high-level of LOH as well as distant metastasis may rely on the tissue type of the cancer's origin. Similarly, a previous study [24] has described that the normal cell of origin is a strong determinant of metastatic spread, because distinct tissue types show distinct metastatic tendencies.

**Figure 5:**
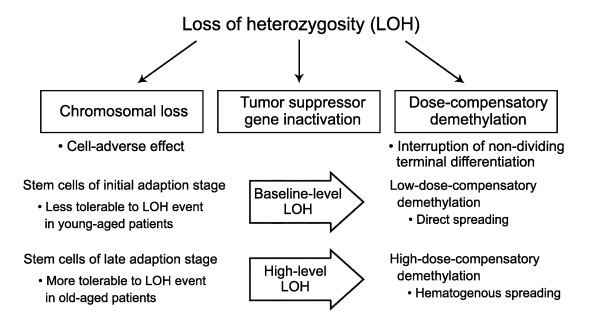
**Schematic diagram of the LOH-related genetic and epigenetic alterations that underlie gastric carcinogenesis**. The general paradigm is that the LOH events exert an adverse effect on cell proliferation and they induce a dose-compensatory response in addition to a key function of the second hit that targets cancer-associated genes. Newly fixed stem cells are increasingly resistant to the adverse effect of LOH events during long-term adaptation to the gastric mucosa, since stem cells establish the high expression of stomach-specific genes that is restricted to some chromosomes. A baseline-level LOH is common in the diffuse-type cancers of young-aged patients at the initial stage of the adapting stem cells. A high-level LOH has a minimal adverse effect on stem cells at a late adaptation stage in old gastric cancer patients who harbor a few highly expressed genes on cancer-associated chromosomes. The dose-compensatory demethylation can lead to the interruption of terminal differentiation and to the persistent expansion of migratory stem cells. The extensive high-dose demethylation leads to the persistent expansion of lineage-committed migratory cells even in distant foreign tissue-environments.

In addition to the cell-adverse effect of LOH, dose-compensatory demethylation provides a plausible explanation for a tissue-type-dependent metastasis tendency. In high-level LOH gastric cancers, the extensively demethylated CpG-island genes can protect disseminated gastric cancer cells from the overmethylation of the CpG-island genes that induce non-dividing terminal differentiation in an unfamiliar tissue environment (Figure 5). A high frequency of the high-level LOH cases in the intestinal and mixed type of gastric cancers implicates a dedifferentiation effect on the intestinal differentiation (Figure 2). Meanwhile, in the baseline- and low-level LOH gastric cancers that show a few cases of disease relapse with distant metastasis, the demethylation of CpG-island genes appears to be insufficient to protect cancer cells from the terminal differentiation induced with the overmethylation of the CpG-island genes. Therefore, it is likely that a high-level LOH resulting from a mild adverse effect of LOH in the stomach tissue environment drives the growth of a micrometastasis into a macroscopic metastasis via high-dose-compensatory demethylation.

The diffuse-type gastric cancers showed a bimodal distribution for the number of chromosomal losses in consistent with a previous study [3]. In a bimodal distribution, there were one and four chromosomal losses that occur frequently. The high-level LOH induced demethylation might lead to the dedifferentiation of gastric cancer cells that were initially well-differentiated. Meanwhile, the baseline-level LOH and low-level LOH cases having a few LOH events are likely to preserve the differentiation state formed in cancer progenitor cells. This implies that the histologic type of a given gastric cancer is variously determined according to the adaptive differentiation of cancer progenitor cells as well as the cell-adverse effect of LOH. In the baseline- and low-level LOH gastric cancers that showed a few cases of disease relapse with distant metastasis, the demethylation of CpG-island genes appears to be insufficient to protect cancer cells from the terminal differentiation induced with the overmethylation of the CpG-island genes.

The MSI-positive cases were oldest among the four genotype groups; baseline-, low-, and high-level of LOH and MSI. Although the gastric cancer patients with low-level and high-level LOH were older than the baseline-level LOH cases that were youngest among the four genotype groups, both the low-level and high-level LOH cases were younger than the MSI-positive cancer patients. The result of this study agrees with the previous studies reporting that the MSI-positive cases were common in late-onset gastric cancers when compared with the LOH-positive cases [7,25]. The gastric cancer with MSI genotype has been known to undergo the hypermethylation of multiple CpG-island genes involving the mismatch repair gene, which is associated with the aging process [12,13,26]. Because the LOH-positive cases may be influenced by the adverse effect of LOH on the cancer progenitor cells, the onset age of gastric cancers is associated distinctly with the LOH and MSI events.

Comparison of the LOH and DNA-copy-number analyses has shown that the level of LOH is underestimated with DNA-copy-number analysis due to the coincidence of chromosomal losses and gains that involve the same genomic position [27]. The complicated chromosomal changes and intra-tumoral heterogeneity have raised a difficult question about which genetic alteration plays a main role in cancer initiation and progression. Multifocal microsatellite analyses of the heterogeneous tissue sites of gastric cancers [3,7,13], sarcomatoid carcinoma [28] and glandular-neuroendocrine carcinoma [29] have demonstrated that the distinct cell components always harbor the same LOH involving the same allele on the cancer-associated chromosomes. These results suggest that the primary chromosomal loss and secondary chromosomal gain direct the subclonal expansion of heterogeneous cancer cells and that chromosomal losses are not exactly compensated with chromosomal gains in view of a dosage compensation.

Dose-compensatory demethylation in response to the LOH event may up-regulate the CpG-island genes containing CpG islands as well as down-regulate the stomach-specific genes lacking CpG islands. This is because the housekeeping genes and the tissue-specific genes share the limited amount of nuclear proteins in a nuclear space and there is an inverse correlation between the expression of the two gene groups [21]. However, it is methodologically difficult to define the accurate expression of the house-keeping genes since most of the up-regulated housekeeping genes are weakly active [11,30]. The methylation status of individual genes is too heterogeneous in heterogeneous tumor sites to be accurately estimated with small pieces of biopsy tissue [13]. In this regard, microsatellite analysis is the method of choice for making the pretreatment genetic diagnosis of gastric cancer.

The pyrosequencing analysis of single nucleotide polymorphism (SNP) is useful to detect LOH [31], but it is not suitable for MSI detection. Highly repetitive microsatellite sequences are useful to analyze the LOH as well as MSI. The radioisotope-labeling PCR protocol used in this study is an advantageous method to obtain the specific PCR bands from a small amount of biopsy tissues under a stringent condition of minimal PCR cycles. The genetic instability of cancer that leads to intratumoral heterogeneity is a difficult problem to be solved for making an accurate pretreatment genetic diagnosis with using a small piece of biopsy tissue. A series of radioisotope-labeled microsatellite analyses of gastric cancers have reported that the level of LOH and MSI are closely related with the clinicopathologic characteristics and survival rate of the gastric cancers [3,4,7]. Other non-radioisotope methods are often problematic for analyzing microsatellite sequences due to a high number of PCR cycles and the simple nucleotide sequences that limit designing specific PCR primers.

## Conclusions

Although the surgical pathology has been used as the gold standard, it remains difficult to identify, before surgical resection, the gastric cancer patients who will have relapsed disease [32]. This study illustrated two distinct high-risk genotypes, high-level and baseline-level LOH. The high-level LOH cases recurred through the hematogenous route after R0 resection of pathologically non-metastatic tumors. The baseline-level LOH cases with early-onset and diffuse-type cancers recurred in the peritoneal cavity rather than in the distant organs. The LOH events combined with a cell-adverse effect and a dose compensatory response in addition to tumor suppressor gene inactivation may activate the stem-cell intrinsic program and this may determine the due clinical course of gastric cancers before tumor cells appear as a tumor mass in situ.

## Competing interests

The authors declare that they have no competing interests.

## Authors' contributions

SJH and EJJ conceptualized, helped with data collection and analysis, and drafted the manuscript. SJH and EJJ contributed equally to this work. JHO helped with performing the endoscopic biopsy and data collection. EJS helped with performing the histopathologic examination. SWC conceived the study, participated in its design and contributed to the manuscript. MGR conceptualized, edited the manuscript for important intellectual content and has read and approved the final version of the manuscript. All authors read and approved the final manuscript.

## Pre-publication history

The pre-publication history for this paper can be accessed here:

http://www.biomedcentral.com/1471-230X/10/138/prepub
